# Magnitude and Contributing Factors of Low Back Pain among Long Distance Truck Drivers at Modjo Dry Port, Ethiopia: A Cross-Sectional Study

**DOI:** 10.1155/2019/6793090

**Published:** 2019-09-22

**Authors:** Tewodros Yosef, Ayele Belachew, Yifokire Tefera

**Affiliations:** ^1^Department of Public Health, College of Medicine and Health Sciences, Mizan-Tepi University, Mizan Teferi, Ethiopia; ^2^School of Public Health, College of Health Sciences, Addis Ababa University, Addis Ababa, Ethiopia

## Abstract

**Background:**

Low back pain (LBP) is well known as the most common musculoskeletal disorder with the lifetime prevalence of eighty percent. Worldwide, 37% of low back pain was attributable to occupational risk factors. Truck driving is one among the jobs causing occupational LBP. Even though these drivers in Ethiopia run the high risk of occupational injuries and illnesses like drivers elsewhere, the evidence that shows the magnitude and factors that contribute to LBP is a significant shortcoming.

**Objective:**

To assess the magnitude and contributing factors of low back pain among long-distance truck drivers at Modjo Dry Port, Ethiopia, 2018.

**Methods:**

A cross-sectional study was conducted among systematically selected 422 long-distance truck drivers at Modjo Dry Port, Ethiopia, from February to March 2018. Data were collected through face-to-face individual interview using a structured questionnaire adapted from the standardized Nordic questionnaire for the analysis of musculoskeletal symptoms. The data were entered using EPI-DATA version 4.2.0.0 and cleaned and analyzed using SPSS version 20 statistical software for windows. Binary logistic regression was computed to determine the association using crude and adjusted odds ratios at 95% confidence intervals. Independent variables with a *P* value less than 0.05 in the multivariable logistic regression model were considered as significant.

**Results:**

Of 400 truck drivers interviewed, the prevalence of LBP was found to be 65%. The study also found smoking cigarette (AOR = 2.24, 95% CI (1.25–4.01), and *P*=0.007), physical inactivity (AOR = 2.12, 95% CI (1.28–3.51), and *P*=0.003), chronic diseases other than LBP (AOR = 2.18, 95% CI (1.32–3.61), and *P*=0.002), frequent lifting or carrying heavy objects (AOR = 3.02, 95% CI (1.75–5.22), and *P* ≤ 0.001), perceived improper sitting posture while driving (AOR = 2.20, 95% CI (1.35–3.60), and *P*=0.002), and perceived job stress (AOR = 2.07, 95% CI (1.20–3.57), and *P*=0.009) were contributing factors of low back pain.

**Conclusion:**

This finding shows the public health importance of low back pain among long-distance truck drivers in Ethiopia. Individual factors largely accounted for the development of low back pain; hence, orientation on these modifiable risk factors and regular follow-up on safety procedures should be considered to mitigate the problem.

## 1. Introduction

Low back pain (LBP) is well known as the most common musculoskeletal disorder with the lifetime prevalence of 80%. However, its prevalence varies across the studied populations, geographic areas, and age groups [1, 2]. LBP is a serious occupational disease and leads to a serious social problem, huge workers' compensation, and a decline in productivity [3, 4]. It is the leading cause of disability globally [5].

Worldwide, 37% of low back pain was deemed attributable to occupational risk factors and the magnitude was generally higher in those regions with lower overall health status [6]. The economic, societal, and public health effects of LBP appear to be increasing. It incurs billions of dollars in medical expenditures each year [7]. The total cost of work-related low back pain monotonically increased in recent years, which entails a considerable economic burden of society [8].

Truck driving is among jobs causing occupational LBP [9]. According to researchers and safety analysts, truck driving is ranked as one of the most dangerous occupations in the world [10]. Truck drivers (TDs) are exposed to stressful working (and living) conditions and are vulnerable. As a result, they face physical and mental health problems and psychological distress more frequently than the general population as a consequence of long driving shifts, disrupted sleep patterns, chronic fatigue, social isolation, delivery urgency, and job strain [11, 12]. Long-distance truckers have some of the highest rates of injuries and illness of all occupations. Around a third of truck drivers in the USA will be involved in a serious road accident at some point during their careers and experiencing potentially severe job-related trauma. Being in a wreck or even seeing someone can cause enough stress and anxiety to become a diagnosable mental illness, such as acute stress disorder or post-traumatic stress disorder (PTSD) [13].

Occupational driving has often been associated with a high prevalence of back pain. A case-control study among 346 truck drivers and office workers in Iran revealed a one-year prevalence of low back pain was 24.3% in truck drivers and 12.1% in official workers [14]. Cross-sectional studies were conducted worldwide and reported that 60% of truck drivers in the UK [15], 59% in Sao Paulo, Brazil [16], 73.5% in India [17], 88.7% in Tanzania, Dares Salaam [18], and 62.1% in Nagpur, India [9], complain of low back pain.

The factors that contribute to cause the pain are diverse and might include work duration, prolonged sitting position, physical workload, lifting or carrying heavy objects, prolonged uncomfortable postures while driving, exposure to whole-body vibration, poor diet, job dissatisfaction and other psychological factors, low socioeconomic status, body mass index, and age [3, 14–16, 19–24].

It is known that truck drivers travel long duration without having adequate resting time [15, 16, 19, 21]. This makes truck drivers present 2.3 times the risk for LBP compared with individuals involved in other occupational activities [25]. Generally, LBP is common among long-distance truck drivers, resulting in reduced health-related quality of life [26]. Making routine stretching habits, using legs for heavy lifting, adjusting the seat, and eating nutritious foods are simple methods that dramatically improve a trucker's back pain [27].

Even though these drivers in Ethiopia run the high risk of occupational LBP like drivers elsewhere, the evidence that shows the magnitude and factors that contribute to LBP is a significant shortcoming. The significance of this study was aimed at estimating the magnitude and assessing the possible contributing factors of low back pain among long-distance truck drivers in order to fill the knowledge gap, and the findings will help policymakers to prioritize action aimed at risk reduction and also provide opportunities for future studies to fill in the gaps that this study could not address.

## 2. Materials and Methods

### 2.1. Study Design, Period, and Setting

A dry port-based cross-sectional study was conducted from February 1 to March 1, 2018, at Modjo Dry Port. Modjo Dry Port is the first dry port established at the end of 2009 to relieve the congestion in the Djibouti port. It is located in central Ethiopia, 38 miles southeast of Addis Ababa. The port handles 95% of Ethiopia's trade and the major bottleneck on the Ethiopia-Djibouti trade corridor. Based on the information gathered from the Modjo Dry Port management authority, approximately around 300–400 trucks arrived every day from the Djibouti port.

### 2.2. Study Population

All truck drivers driving between Djibouti International Port and Modjo Dry Port were the source population. Drivers who had at least one-year experience were included in the study to gain the minimum exposure time. However, drivers above 60 years of age were excluded to control age-related effect and those who had a history of accident from a known cause such as car crash and fall were also excluded since they are at risk for developing LBP from the trauma.

### 2.3. Study Variables

The dependent or response variable was low back pain. The independent or predictor variables were sociodemographic characteristics (age in completed years, education, monthly income, marital status, family size, weight, height, and BMI), lifestyle (smoking cigarettes, chewing chat, drinking alcohol, and physical exercise), medical history (diseases other than LBP), work characteristics (average daily driving hours, years spent driving, rest breaks between driving, and frequent involvement in lifting or carrying of objects), and psychological factors such as work stress, job satisfaction, and adequacy of spending time with family.

### 2.4. Sample Size Determination

The sample size was determined using a single population proportion formula with the input of *p* = expected proportion of truck drivers with low back pain (50%), 5% precision level, 95% confidence interval, and 10% nonresponse compensation. Using the following formula, the sample size computed was 422:

(1) $$\begin{array}{c} n=\frac{{\left(Z\alpha /2\right)}^{2}p\left(1-p\right)}{d^{2}}, \end{array}$$

where *n* is the sample size, *p* is the expected proportion of truck drivers with low back pain, *d* is the margin of error (precision level), and *Z* *α*/2 is the reliability coefficient (confidence coefficient).

### 2.5. Sampling Method

On average, a maximum of 15 days is required for a truck to make a round trip from Modjo Dry Port to Djibouti International Port and back to Modjo Dry Port unless a technical problem on the vehicle or other accidents occurred. Based on the information from the port management, an average of 300 to 400 trucks arrives daily at the port. With this consideration to give each driver an equal chance of inclusion, the total sample size was divided by fifteen days and concluded that 28 truck drivers can be studied every day. To identify the potential study participants using the systematic random sampling technique, 300 was divided by 28 to obtain the constant for the sampling interval, which was 11. A random number between one and eleven was chosen as a starting number; in this case, it was 6. Hence, every eleventh driver from the 6^th^ driver was studied until the total sample size was obtained.

## 3. Operational Definitions

- Low back pain (LBP) was defined as pain at the lower back of the body at the time of driving or after having a long-time driving
- The prevalence of LBP is the frequency of study subjects who will respond to experiences of LBP in the past twelve months' time
- A smoker is a person who smokes cigarettes daily whatever the number of cigarettes
- Drunker is a person who drinks beer, local beer or areke, tella, or tej every day or every other day
- Chat chewer is a person who chews chats at least once within a week
- Having physical exercise means if someone had 3 or more days of physical exercise (walking, running, bicycling, and stretching exercise such as sit-ups and pull ups)
- Rest breaks between driving means if a driver takes rest after an hour or more of driving but not included rest for a meal (at restaurants and cafeterias found on the way with a reasonable distance)
- Improper seating posture means if someone had bending, twisting, and half-buttock sitting posture while driving
- Frequent involvement in carrying or lifting heavy objects means a driver who involved in carrying or lifting objects weighing 25 and above kilograms every day or every other day

### 3.1. Data Collection Instrument and Procedures

A modified standardized Nordic questionnaire for the analysis of musculoskeletal symptoms [28] was used. Data were collected through face-to-face interviews. And anthropometry (weight and height) was calculated using a calibrated scale to compute the body mass index of the study participants. The questionnaire was composed of five sections: sociodemographic factors, lifestyle and medical factors, LBP and pain characteristics, ergonomic/work characteristics, and psychosocial factors. First, the questionnaire was prepared in English, translated into the local language (Amharic), and then retranslated into English to maintain its consistencies. A three-day training was given for data collectors and supervisors concerning the objective and the process of data collection and discuss the presence of an ambiguous question in the questionnaire and, if there, it was clarified. Finally, an occupational health specialist checked the validity of the tool, and a pilot study was done on 5% of the sample size in the Akaki area, in which trucks were densely found. The data were collected by four qualified BSc environmental and occupational health professionals, and two qualified MPH professionals supervised the data collection and its completeness.

### 3.2. Data Processing and Analysis

The collected data were coded, entered using EPI-data version 4.2.0.0, cleaned, and analyzed using SPSS version 20 statistical software. Summary statistics of independent variables were presented using frequency tables. Binary logistic regression was computed to determine the association using crude and adjusted odds ratios at 95% confidence intervals. Independent variables found significant with a *P* value less than 0.05 at the bivariate level were included in the multivariable binary logistic regression model to control for potential confounding. Multicollinearity between exposure variables was checked. The Hosmer–Lemeshow goodness of fit test was performed to check model adequacy.

## 4. Results

Out of 422 respondents, four hundred male drivers participated in this study, giving a response rate of 94.8%.

### 4.1. Sociodemographic Characteristics of Truck Drivers

The mean age of the respondents was 37.7 (±9.13 SD) years with a range of 22 to 59 years. Two hundred ninety-two (73%) of the respondents were Orthodox by religion. Two hundred sixty-eight (67%) were married, and two hundred sixty-seven (66.8%) achieved secondary school. Their mean monthly income was 220 (±91) USD, and the median monthly income was 198 USD, ranging from 74 to 741 USD. One hundred forty-nine (48.5%) respondents were overweight (Table 1).

### 4.2. Lifestyle and Medical Characteristics of Truck Drivers

One hundred twenty-four (31%) respondents habitual smokers, and one hundred thirty-nine (34.8%) were chatting chewers. Two hundred sixty-four (66%) were alcohol drinkers. Two hundred fifty-five (63.8) had no habit of regular physical activity. One hundred ninety-six (49%) had less than 6 hours of sleep daily. One hundred sixty-seven (41.8%) had chronic diseases other than LBP (Table 2).

### 4.3. Magnitude and Characteristics of Low Back Pain of Truck Drivers

Two hundred sixty (65%) of the drivers reported low back pain at least once in the last twelve months. Out of those with LBP, for two hundred six (79.2%) the type of pain was self-limiting without any treatment. The pain among two hundred twenty-one (85%) was severe, while in thirty-seven (14.2%) and one hundred sixty (61.5%), the pain was chronic and spread to the lower part of the body (leg and buttock), respectively (Table 3).

### 4.4. Occupational and Ergonometric Characteristics of Truck Drivers

The mean daily driving hours were 11.5 (±2.76 SD) hours with a range of 6 to 18 hours. Two hundred fifty-three (63.2%) reported driving an average of 10–15 hours on a typical day. Three hundred thirty-nine (84.8%) had broken in between driving, of this 51.3% for thirty and more minutes. Three hundred eight (77%) drivers were frequently involved in lifting or carrying objects (25 and above kilograms). Regarding sitting posture, two hundred twenty-two (55.5%) perceived that they had a “proper” sitting posture while driving. Two hundred sixteen (54%) reported the absence of suspension seats on their trucks. Two hundred ninety-six (74%) reported the presence of adjustable back support on their trucks (Table 4).

### 4.5. Psychosocial Characteristics of Truck Drivers

Two hundred forty-nine (62.2%) reported that they are satisfied with their job while three hundred three (75.8%) perceived that their job is stressful. Two hundred ninety-seven (74.2%) and three hundred thirty-nine (84.8%) had boredom with the job and short spending time with their family, respectively.

### 4.6. Bivariate and Multivariate Analyses

Bivariate analysis was done for potentially expected contributing factors. To avoid overfitting in the final model because of an excessive number of variables, only variables found statistically significant at *P* < 0.05 in the bivariate analysis and variable not statistically significant but had clinical importance (average daily driving hours) were included in the multivariable binary logistic regression model. Multicollinearity between independent variables in the model was checked, and the variance inflation factor (VIF) was found acceptable (less than 2). The Hosmer–Lemeshow goodness of fit test indicated (*P*=0.717) that the model was good enough to fit the data well. Finally, smoking cigarette, physical inactivity, chronic diseases other than LBP, frequent involvement in lifting or carrying heavy objects, perceived improper sitting posture while driving, and perceived job stress were significantly associated with low back pain (Table 5).

## 5. Discussion

This study aimed to assess the magnitude and contributing factors of low back pain among long-distance truck drivers at Modjo Dry Port. As a result, the twelve months' prevalence of low back pain among long-distance truck drivers was found to be 65%. It was higher than 62.1% prevalence rate in Nagpur, India [9], 60% in the UK [15], 59% in Sao Paulo, Brazil [16], and 24.3% in Iran [14], but lower than 72.5% in the USA [29], 73.5% in India [17], and 88.7% in Dar es Salaam, Tanzania [18]. The variation observed compared with other studies could be due to the differences in methodology, sample size, and road infrastructure because most of the high prevalence rates indicated above were calculated with low sample size relative to the low prevalence rate.

The current scientific knowledge proved that physical inactivity is associated with several disease conditions. As a result, science recommends a person should have at least three days of physical activity (thirty minutes in a day) per week. But more than half of the drivers have had no habit of physical activity (they had less than three days of physical exercise). Owing to this, the higher odds of acquiring LBP were seen among physical inactive drivers in this study. Being physically inactive was very strongly associated with LBP. This finding is consistent with previous studies done in Israel and Italy [4, 25, 30].

The increased odds of developing low back pain were observed among drivers with chronic diseases other than LBP. Having chronic diseases other than LBP was highly associated with LBP. This finding was supported by Miyamoto et al. [19]. This could be because chronic disease patients had also a concomitant vascular and neural disease. This study also showed the association of cigarette smoking and low back pain explained by the mechanism of injury in low back pain is damage to the vascular structures of the discs and joints as a result of smoking cigarette.

More than three-fourths of the drivers were frequently involved in lifting or carrying objects. Frequent involvement in lifting or carrying objects was strongly associated with an increased risk of developing LBP. It could be due to improper lifting or carrying activities (bad ergonometric) that are prone to back injury. Since lifting objects improperly or carrying objects not balanced with their capacity may result in trauma to the back and finally back pain. This finding was similar to previous studies done in the UK and Addis Ababa, Ethiopia [15, 22].

The odds of developing LBP among drivers with perceived improper seating posture while driving were higher compared with drivers with perceived proper seating posture while driving. Improper seating such as half buttock sitting, bending forward to the steering wheel, and lateral twisting either to the window of the truck's door or in the opposite direction of it while driving can cause compression on the lumbar structure and finally result in LBP. The finding was supported by studies conducted in Malaysia [21, 23]. A consistent result [20] was revealed in this study regarding the association between perceived job stress and LBP. It could be due to stress that activates the body's stress response, which creates a cascade of chemical changes in the body, which in turn leads to muscle tension, muscle spasm, and consequent low back pain.

## 6. Conclusion

The twelve month' prevalence of LBP among long-distance truck drivers was found remarkable (65%), and we can conclude that LBP is the public health importance among truck drivers in Ethiopia. Individual factors were largely accounted for the development of low back pain; hence, orientation on these modifiable risk factors and regular follow-up on safety procedures should be considered to mitigate the problem.

## Figures and Tables

**Table 1:** Sociodemographic characteristics of the respondents at Modjo Dry Port, Ethiopia, 2018.

Variables	Frequency (*n*)	Percent
Age group (year)		
Below 37.7 years	203	50.8
Above 37.7 years	197	49.2

Religion		
Protestant	33	8.2
Orthodox	292	73
Muslim	75	18.8

Marital status		
Single	96	24
Married	268	67
Separated/divorced/widowed	36	9

Level of education		
Read and write up to grade 8	92	23
Grade 9–12	267	66.8
College or university	41	10.2

Income		
Below 220 USD	204	51
Above 220 USD	196	49

Body mass index (BMI in kg/m^2^) category		
18.5–24.9	174	43.5
25–29.9	194	48.5
30 and above	32	8

**Table 2:** Lifestyle and medical characteristics of the respondents at Modjo Dry Port, Ethiopia, 2018.

Variables	Frequency	Percent
Smoking cigarette		
Yes	124	31
No	276	69

Alcohol drinking		
Yes	264	66
No	136	34

Chat chewing		
Yes	139	34.8
No	261	65.2

Physical exercise		
Yes	145	36.2
No	255	63.8

Hours took for sleeping		
<6 hours	196	49
≥6 hours	204	51

Diseases other than LBP		
Present	167	41.8
Absent	233	58.2

**Table 3:** The magnitude and characteristics of low back pain in the last 12 months among respondents at Modjo Dry Port, Ethiopia, 2018.

Variables	Frequency (*n*)	Percent
Presence of LBP (*n* = 400)		
Yes	260	65
No	140	35

Nature of pain (*n* = 260)		
Self-limiting without treatment	206	79.2
Continuous without treatment	54	20.8

Level of pain at episode (*n* = 260)		
Mild	106	40.8
Moderate	117	45
Severe	37	14.2

Duration of LBP (*n* = 260)		
Acute (<6 weeks)	10	3.8
Subacute (6–12 weeks)	29	11.2
Chronic (>12 weeks)	221	85

Spread of LBP to the lower part of the body (*n* = 260)		
Yes	160	61.5
No	100	38.5

**Table 4:** Occupational and ergonometric risk factors among respondents at Modjo Dry Port, Ethiopia, 2018.

Variables	Frequency (*n*)	Percent
Years spent truck driving		
<10 years	211	52.8
≥10 years	189	47.2

Average daily driving hours		
<10 hours	100	25
10–15 hours	253	63.2
>15 hours	47	11.8

Rest breaks between driving		
Present	339	84.8
Absent	61	15.2

Frequent involvement in lifting/carrying heavy objects		
Yes	308	77
No	92	23

Perceived sitting posture while driving		
Proper	222	55.5
Improper	178	44.5

Truck with suspension seat		
Yes	184	46
No	216	54

Truck with adjustable back support		
Yes	296	74
No	104	26

**Table 5:** Bivariate and multivariate logistic regression analyses of factors associated with low back pain among respondents at Modjo Dry Port, Ethiopia, 2018.

Variables	Presence of LBP		COR (95% CI)	AOR (95% CI)	*P* value
	Yes, *n* (%)	No, *n* (%)			
Age group (years)					
<37.7 years	112 (55.2%)	91 (44.8%)	1.00	1.00	
>37.7 years	148 (75.1%)	49 (24.9%)	2.45 (1.60–3.76)^*∗*^	1.37 (0.72–2.60)	0.342

Smoking cigarette					
Yes	103 (83.1%)	21 (16.9%)	3.72 (2.20–6.29)^*∗*^	2.24 (1.25–4.01)	0.007^*∗*^
No	157 (56.9%)	119 (43.1%)	1.00	1.00	

Physical exercise					
Yes	76 (52.4%)	69 (47.6%)	1.00	1.00	
No	184 (72.2%)	71 (27.8%)	2.35 (1.54–3.60)^*∗*^	2.12 (1.28–3.51)	0.003^*∗*^

Hours took for sleeping					
<6 hours	142 (72.4%)	54 (27.6%)	1.92 (1.26–2.91)^*∗*^	1.26 (0.76–2.07)	0.373
≥6 hours	118 (57.8%)	86 (42.2%)	1.00	1.00	

Chronic diseases other than LBP					
Present	130 (77.8%)	37 (22.2%)	2.78 (1.78–4.36)^*∗*^	2.18 (1.32–3.61)	0.002^*∗*^
Absent	130 (55.8%)	103 (44.2%)	1.00	1.00	

Years spent truck driving					
<10 years	114 (54%)	97 (46%)	1.00	1.00	
≥10 years	146 (77.2%)	43 (22.8%)	2.89 (1.87–4.46)^*∗*^	1.66 (0.86–3.19)	0.129

Average daily driving hours					
<10 hours	60 (60%)	40 (40%)	1.00	1.00	
10–15 hours	165 (65.2%)	88 (34.8%)	1.25 (0.78–2.01)	1.01 (0.58–1.76)	0.978
>15 hours	35 (74.5%)	12 (25.5%)	1.94 (0.90–4.19)	1.61 (0.65–4.03)	0.306

Frequent lifting or carrying objects					
Yes	220 (71.4%)	88 (28.6%)	3.25 (2.01–5.26)^*∗*^	3.02 (1.75–5.22)	≤0.001^*∗*^
No	40 (43.5%)	52 (56.5%)	1.00	1.00	

Perceived sitting posture while driving					
Proper	125 (56.3%)	97 (43.7%)	1.00	1.00	
Improper	135 (75.8%)	43 (24.2%)	2.44 (1.58–3.76)^*∗*^	2.20 (1.35–3.60)	0.002^*∗*^

Perceived job stress					
Yes	212 (70%)	91 (30%)	2.38 (1.49–3.80)^*∗*^	2.07 (1.20–3.57)	0.009^*∗*^
No	48 (49.5%)	49 (50.5%)	1.00	1.00	

CI = confidence interval; COR = crude odds ratio; AOR = adjusted odds ratio; ^*∗*^significant at *P* value <0.05.

## Data Availability

The data set is handled by the corresponding author and can be provided upon request.

## Abbreviations

AOR: Adjusted odds ratio
CI: Confidence interval
COR: Crude odds ratio
LBP: Low back pain
SPSS: Statistical Package for the Social Sciences
SD: Standard deviation
UK: United Kingdom
USA: United States of America.

## Additional Points

*Limitations*. Failing to use clinical signs and symptoms to diagnose LBP and failing to use measurement tools for some variables (such as job satisfaction, job stress, and proper sitting posture).
